# Supplementary material for: Identification and functional analysis of non-coding regulatory small RNA FenSr3 in Bacillus amyloliquefaciens LPB-18
Source: PeerJ. 2023 May 15;11:e15236. doi: 10.7717/peerj.15236 (PMC10194069; doi:10.7717/peerj.15236)
Supplement: Supplemental Information 4 [file peerj-11-15236-s004.zip › KO/CK-vs-T1_map/map04723.html]

KEGG PATHWAY: Retrograde endocannabinoid signaling - Reference pathway


|  |  |
| --- | --- |
| **Retrograde endocannabinoid signaling - Reference pathway** |  |

[
Pathway menu
| Organism menu
| Pathway entry
| Show description
| User data mapping
]

Endogenous cannabinoids (endocannabinoids) serve as retrograde messengers at synapses in various regions of the brain. The family of endocannabinoids includes at least five derivatives of arachidonic acid; the two best characterized are arachydonoyl ethanolamide (anandamide, AEA) and 2-arachydonoil glycerol (2AG). They are released from postsynaptic neurons upon postsynaptic depolarization and/or receptor activation. The released endocannabinoids then activate the CB1 receptors (CB1R) at presynaptic terminals and suppress the release of inhibitory transmitter GABA (depolarization-induced suppression of inhibition, DSI) or excitatory transmitter glutamate (depolarization-induced suppression of excitation, DSE) by inhibiting Ca2+ channels. Besides the well-known expression of the CB1R in the plasma membrane, this receptor is also present in mitochondrial membranes, where it reduces the mitochondrial respiration and contributes to DSI. Whereas DSI and DSE result in short-term synaptic plasticity, endocannabinoids also mediate long-term synaptic changes (eCB-LTD). Persistent activation of CB1 receptors over a period of minutes triggers eCB-LTD by a RIM1alpha-dependent mechanism.

|  |  |  |
| --- | --- | --- |
| Reference pathway | 184% 150% 122% 100% 82% 67% 55% | 图片下载 |
